# Global indicators framework for socially responsible research and innovation (RRI): Aligning standards to monitor public and researcher perspectives with the UNESCO Recommendation on Science and Scientific Researchers

**DOI:** 10.12688/openreseurope.14232.2

**Published:** 2023-08-02

**Authors:** Eric A. Jensen

**Affiliations:** 1 ICoRSA, Cork, Ireland; 2 University of Warwick, Coventry, UK; 3 Scotland's Rural University College, Aberdeen, UK

**Keywords:** RRI, socially responsible science, researchers, scientists, indicators, evaluation

## Abstract

As calls for more socially responsible research and innovation (RRI) policies and practices grow more insistent, the need for high-quality indicators that can be used to evaluate progress is becoming increasingly important. Given the global nature of science, such indicators need to be relevant to countries across all world regions. Moreover, the methodological quality of indicators is critical to provide a strong foundation for long-term comparative measurement of the impacts of different kinds of policy intervention. There is a practical challenge here, given the uneven mechanisms for data collection and analysis available in different countries. There is also a geopolitical challenge in gaining buy-in from countries with very different, and sometimes competing, agendas. Here, the 2017 UNESCO-led Recommendation on Science and Scientific Researchers is highlighted as an existing vehicle that can enable cooperation on globally comparative measurement of socially responsible research and innovation. In particular, the quadrennial monitoring of the implementation of this wide-ranging global policy instrument that has been ratified by 195 countries affords a unique opportunity to add value for these countries by linking RRI to the 2017 Recommendation while establishing benchmark indicators for RRI more generally. As a practical and methodological contribution to the global community of science and innovation policymakers, researchers and research and innovation stakeholders committed to socially responsible research, this report contains detailed survey questions and response options focusing on public opinion and individual researchers’ level of measurement. It provides details of sources of benchmark survey data that have readily available open data that can be used to benchmark the development of socially responsible research and innovation over time from the vantage points of the public and researchers around the world. The aim of this kind of science ecosystem-level indicators framework is to enable evidence-based practice in socially responsible research and innovation.

## Plain language summary

The United Nations is overseeing a global effort to improve scientific research policy and practices. This effort requires good metrics to measure progress. Developing appropriate metrics for good policy and practice in science systems is complicated, especially when trying to align approaches globally. This paper offers ideas about how this measurement task can be achieved at the level of public opinion and individual researchers. The recommended approach saves resources and improves quality by using existing metrics and data.

## Introduction

Responsible research and innovation (RRI) has been a key priority for the European Union for many years. Efforts to advance socially responsible research and innovation aim to get science and technology to take into account societal needs and ethical considerations, while driving engagement and dialogue with relevant parties who may be affected. It involves incorporating public concerns and perspectives into scientific and innovative processes to ensure beneficial outcomes and foster societal acceptance. In practice, RRI involves an insistence that research and innovation work in synchrony with society, addressing its diverse needs, whilst prioritising ethical transparency and inclusivity. It strives to create an ecosystem where science and society co-evolve, with the former respecting the latter's values, aspirations and reservations. In recent years, the need for high quality measurement approaches to evaluate responsible research and innovation policies and practices has become increasingly apparent. Establishing globally relevant and usable indicators for RRI is challenging but essential, given the global nature of science. Ensuring these indicators are methodologically robust is important but must be balanced against practical constraints facing measurement initiatives in this domain ( Jensen, 2020a; Jensen, 2020b; Jensen & Lister, 2015). This is a daunting given the uneven mechanisms for data collection and analysis available in different countries (e.g., Heras *et al.,* 2016; Heras & Ruiz-Mallén, 2017).

The geopolitical challenge of gaining consensus and buy-in from countries with very different and sometimes competing agendas to align their RRI indicators can be addressed in part by tethering those indicators to a shared global policy instrument. Here, the 2017 UNESCO-led Recommendation on Science and Scientific Researchers (RS/SR) ( UNESCO, 2017) is highlighted as a valuable existing vehicle to enable cooperation on globally comparative measurement of socially responsible research and innovation. Numerous aspects of RRI that are enshrined in the RS/SR have implications for public views on the role of science. Therefore, it is worth considering including an indicator dimension that focuses on the public aspect of the RS/SR priority areas. The framework focuses on aligning the public opinion level of measurement to benchmark global survey measures, where feasible, such as the Wellcome Global Monitor (WGM) ( wellcome.org/reports/wellcome-global-monitor-mental-health/2020wellcome.org/reports/wellcome-global-monitor-mental-health/2020).

In particular, the quadrennial monitoring of the implementation of this wide-ranging global policy instrument that has been ratified by 195 countries affords a unique opportunity to add value for these countries by linking RRI to the Recommendation while establishing benchmark indicators for RRI more generally. As a practical and methodological contribution to the global community of science and innovation policymakers, researchers, and research and innovation stakeholders committed to socially responsible research, this essay contains specific, detailed survey questions and response options focusing on the public opinion and individual researcher level of measurement. It provides details of sources of international survey data that are readily available for secondary analysis to benchmark the development of socially responsible research and innovation over time from the vantage point of the public opinion.

10 key priority areas were identified and agreed by UNESCO Member States as a practical way to simplify and focus RSSR implementation and long-term monitoring, given the sprawling coverage of the full policy text (see Jensen, 2022a). This document outlines benchmark survey indicators and concomitant open data sources pegged to these 10 key priority areas that can be used to evaluate the development of RRI principles that are integral to the RSSR policy instrument. This measurement approach also allows for the fact that many of the principles included in the RSSR are already in evidence to some extent at different levels of national research systems, yet public opinion on these topics is not well established.

## Public opinion level of measurement for global RRI indicators

This manuscript goes beyond the current state of the art by presenting a framework of indicators that can be used to gain an understanding of RRI progress at the public level. This public level of measurement is often overlooked in a focus on national statistics and research and innovation *per se*, but the public’s perspective on progress towards ever more socially sustainable science and innovation is essential to the long-term health of research and innovation ecosystems globally. Providing RRI indicators at this level of measurement offers specific ways to improve on existing RRI indicators and better support evidence-based practice in this domain ( Jensen & Gerber, 2020).


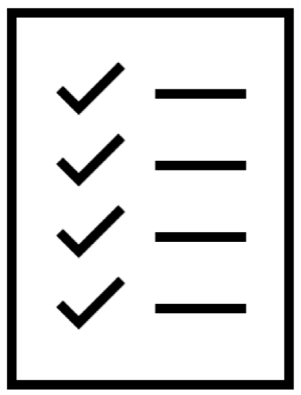
 The checklist icon (left) in this document highlights questions linked directly to the formal national reporting ( UNESCO, 2021) for the UNESCO-led Recommendation on Science and Scientific Researchers RSSR ( UNESCO, 2017). Answers to these overarching questions guide the reader to parts of the document likely to be most relevant. This is designed to highlight relevant RRI measures and indicators that have clear links to the long-term monitoring of the RSSR’s implementation.

Direct quotations from the RSSR are included in grey font in the underlying data ( Jensen, 2022b) to help clarify the different aspects of the categories included in the 10 key priority areas.

Here, indicators are documented that can provide evidence relevant to the public opinion level of responsible research and innovation indicators. Here, these indicators are organised according to the 10 key priority areas for the UNESCO RS/SR. This section of the report describes the sources of global survey data that have been used to provide these indicators.

**Table:** 

Survey	Description	Relevant detail for RS/SR
**Wellcome Global Monitor** **(WGM)**	The only public attitudes to science and health survey conducted on a truly global scale is the Wellcome Global Monitor ( wellcome.org/reports/wellcome-global-monitor-mental-health/2020). wellcome.org/reports/wellcome-global-monitor-mental-health/2020). Crucially, this survey follows good industry standard practices for quality assurance, and it has collected data using probability-based sampling methods from people 15 years or older in over 140 countries. It also gathers demographic data on variables such as education, nationality, gender, and income. The mean number of respondents per country covered is 1,000. The survey was undertaken in 2018 and 2020-21.	This survey covers key variables relevant to the UNESC RS/SR, including public trust in science.
P **ew Research Center** **– International science** **survey questionnaire** **(Pew ISSQ).**	Most recently, 20 countries were covered by this survey in 2019-2020 (age 18+ sample) with a wide geographical spread.	This international science survey of science and society topics includes the extent of public trust in scientists, consumption of science news, views about science policy, and government investment in science, as well as a range of other related topics ( pewresearch.org/science/dataset/international-science-survey).
**World Values Survey** **(WVS)**	The world values survey was conducted between 2017 and 2020 ( worldvaluessurvey.org/wvs.jsp). worldvaluessurvey.org/wvs.jsp). The country coverage s extensive, including 49 nations from a wide range of geographically dispersed regions.	In this dataset, there are relevant survey questions to RRI and the RS/SR, including information about public values relevant to science.
**Special Eurobarometer** **‘public perceptions of** **science, research and** **innovation’**	A special Eurobarometer survey was carried in 2014, focusing on European Union (EU) citizens’ attitudes about scientific research and related issues	This dataset covers public attitudes about science, research and innovation with samples in countries across Europe.
**3M State of Science** **Index Survey**	(). With an average sample size of 1,000 for consistency, this public attitudes survey has been gradually adding additional country coverage over time, and already spans different world regions.	The 3M annual state of science index measures science attitudes in 14 countries ( dataset), focusing on various aspects of public views about science. This dataset covers a relatively small number of countries but offers good coverage for those countries.
**SFI Science in Ireland** **Barometer survey**	An example of a national-level science attitudes survey, this survey was conducted in 2020-2021 in Ireland. It followed robust methodological procedures in the set-up, piloting/validation, and implementation of the survey design..	This survey dataset covers a wide range of RRI-related topics, including public views about science’s inclusiveness, whether the benefits of science are widely shared and gender equality in science.

## Individual researcher level of measurement for global RRI indicators

This essay goes well beyond the state of the art by presenting a framework of indicators that can be used to gain an understanding of RRI progress at the individual researcher level. These indicators are mapped directly to the formal national reporting ( UNESCO, 2021) for the UNESCO-led Recommendation on Science and Scientific Researchers (RSSR) ( UNESCO, 2017). 

This report documents indicators that can provide evidence relevant to the individual researchers’ level of socially responsible research and innovation indicators ( Jensen, 2022b). Here, these indicators are organized according to the 10 key priority areas for the UNESCO RSSR. This section of the report describes the sources of global survey data that have been used to provide these indicators, and that research and science policy stakeholders around the world can use to assess their current status with responsible research and innovation.

**RRING survey on socially responsible research/innovation.** A global survey was launched as a part of the Responsible Research and Innovation Networked Globally (RRING) project ( zenodo.org/communities/rring). The survey was open from 1 ^st^ October 2019 to 20 ^th^ December 2019. Aiming to get a deeper insight into the practices and policies of responsible research and innovation (RRI) across the world, this study was conducted across 20 countries. Diversity was ensured across factors such as the research and development expenditures, per capita income levels, etc., while selecting the countries to prioritise for saturation sampling (a mix of locally organised data collection in selected countries and an overall open call for responses, with email-based participation requests, was used).

The survey gathered 2,198 survey responses (70+% complete) and 539 surveys under 70% complete. Mean completion time for respondents was 33 minutes (dataset available: https://doi.org/10.5281/zenodo.5031585).

**Organisation for Economic Co-operation and Development (OECD) International Survey of Scientific Authors.** As an online survey conducted worldwide, the international survey of scientific authors (ISSA) aims to evaluate science’s development in its use of digital tools ( oecd.org/science/survey-of-scientific-authors.htm). It gathered responses from close to 12,000 scientific authors. The survey was conducted under the auspices of the OECD-organized Working Party of National Experts of Science and Technology Indicators (NESTI) ( https://community.oecd.org/docs/DOC-174112).

The study targeted the corresponding authors of scientific publications whose contact information is available in a large global bibliographic database. A sample of scientific authors listed as corresponding authors received participation requests directly from the OECD and were asked to report on their use of a broad range of digital tools and related practices, in addition to another key demographic and career information. Responses were collected for a total of approximately 12,000 scientific authors from all over the world and across all disciplinary areas, representing to a varying extent the subset of the research population engaged in scholarly publication work, including those in the business sector.

**Frontiers’ Academic response to COVID-19 survey.** Frontiers is an open access research publisher and open science platform ( apo.org.au/node/309304). Frontiers commissioned a survey focusing on how the COVID-19 pandemic has affected the practice of science around the world, using its database of active researchers who have published their research with Frontiers or have acted as reviewers or editors. 

The survey was conducted in May-June 2020. A total of 25,307 respondents from 152 countries answered at least one question and 17,644 completed the entire fully anonymised survey. The 30 countries all had more than 100 respondents and make up 88% of the 17,690 respondents who provided information about their location.

## Conclusion

RRI initiatives strive for research and innovation to chart a course where scientific and technological developments are intrinsically entwined with societal values, needs, and ethical considerations. The purpose is not only to produce knowledge or inventions, but to do so in a way that respects the environment, the wider public, and the fundamental ethical standards that bind us all together. The EU has invested considerable time and resources in promoting socially responsible research and innovation policies and practices over the last decade. This has resulted in ever-greater demand for measurement frameworks and indicators capable of assessing whether this intervention is delivering improvements in research and innovation systems (e.g., Heras *et al.,* 2016; Heras & Ruiz-Mallén, 2017). It is important to scale up RRI evaluation to a global level, moving beyond the limited range of considerations and perspectives that define any one world region’s approach to developing socially responsible research policies and practices. Relevant to this ambition, the UNESCO-led RS/SR ( UNESCO, 2017) establishes formally agreed expectations for national research systems that are well-aligned with RRI principles. Because 195 countries have signed onto this legal instrument, including the requirement for quadrennial monitoring reports, it makes for an excellent vehicle to develop globally relevant RRI indicators. The fact that so many countries are undertaking national assessments relating to RRI on the same timescale, and with the same focus areas, bolsters the potential value of benchmark indicators to be used across multiple countries. Moreover, parallel evaluation of RRI’s integration in national research ecosystems on a global scale creates the opportunity for comparative analysis. Such comparative analysis can help to reveal the interventions that most effectively improve RRI policy and practice globally.

Global monitoring and evaluation of RRI-related outcomes is rife with complicated practical and methodological challenges, given the diversity of priorities, interests, resource levels, and capabilities for undertaking measurement exercises in this domain internationally. RRI indicators should be globally relevant, given that science itself is a global enterprise. In addition, it is vital that indicators deliver precise results because they will be used to determine which kinds of RRI policies and practices are most worthy of further investment and development. Methodological rigour is also important to establish a good basis for comparative analyses ( Jensen, 2020a; Jensen, 2020b; Jensen & Lister, 2015), looking across different research and innovation systems to reveal the factors affecting the efficacy of RRI interventions.

This manuscript indirectly addresses the need for methodologically sound measurement approaches for RRI monitoring and evaluation at the level of individual researchers and public opinion. This is because standardised measurement, applied at a regular interval, will likely deliver higher quality data than isolated one-off efforts. Indeed, appropriate methods of monitoring and evaluation are achievable through the application of relevant social research principles:

“Evaluation is just one type of research framework, which focuses on whether a set of objectives have, in fact, been achieved. There is every reason to expect both knowledge and practical guidance to emerge from the same well-designed impact evaluation.” ( Jensen, 2014: C04)

Beyond the social research aspect of evaluation, however, the practicalities of implementing an indicators framework inevitably affect methodological rigour. Who will ensure that indicator data are collected? How will the quality of that data be assured? What will motivate nations or organisations to collect, collate, analyse and report such data? This manuscript offers an indicators framework that comes with practical solutions to these challenges for addressing the public opinion and individual researcher level of measurement ( Jensen, 2022b). The proposed use of a range of relevant data sources containing global researcher survey data provides an opportunity for methodological triangulation between different surveys and question types. These international survey data sources (and others) can be used to compare results between countries with the aim of identifying good practices worthy of wider implementation.

If the type of international surveys noted in this essay are used, then opportunities for useful empirical insights emerge that can reflect back on RRI’s conceptualisation itself. To effectively mobilise evidence-based practice in the context of RRI, a wider community of international research policy, practice and evaluation must emerge to bring diverse perspectives into dialogue (e.g., see Jensen & Gerber, 2020). Such efforts can greatly enrich the RRI monitoring and evaluation process by taking into account the different socio-historical and cultural characteristics of each country.

The rationale for introducing high quality public indicators to monitor RRI globally is to enable evidence-based practice in the field of socially responsible research and innovation ( Jensen, 2014; Jensen & Gerber, 2020). Without high-quality measurement in place ( Jensen & Laurie, 2016), it is difficult to accurately pinpoint the policies that are effective in developing healthier research and innovation ecosystems (cf., Lindner, 2016). Moreover, flying blind without such measurement increases the risk that well-intentioned but ill-conceived RRI policy and practice interventions that have a negative impact in practice go undetected, unchecked and unreformed.

This manuscript provides a concrete proposal of survey questions that can be used to measure key aspects of socially responsible research and innovation at the level of public opinion. This level of measurement is important, given that the public ultimately provides funding and political support for long-term research and innovation funding from the government, and should be a major downstream beneficiary of research and innovation. The advantage of these particular questions is that they already have data available across many countries and/or have been put through methodological testing to ensure that they are robust. These survey questions are being offered up to start a wider conversation about how best to evaluate progress in the development of healthy and socially responsible research systems. Further development and refinement of the framework presented in this essay will certainly be needed. The long-term journey towards socially responsible research and innovation systems will benefit from such improvements to monitoring and evaluation methods.

## Ethics and consent statement

Ethical approval and consent were not required for the aspect of the RRING (rring.eu) project this paper is part of. However, the project as a whole was given ethical clearance by the University College Cork institutional review board.

## Data Availability

Zenodo Global indicators framework for socially responsible research and innovation (RRI): How to monitor public and researcher perspectives (Supplemental material). https://doi.org/10.5281/zenodo.5886044. ( Jensen, 2022b). This project contains the following underlying data: Supplementary materials: Detailed guidance on benchmark survey questions for evaluating socially responsible research progress at the level of public opinion. (This dataset provides a detailed account of indicators that can be used to measure RRI progress around the world against the UNESCO Recommendation for Science and Scientific Researchers at the level of public opinion.). Supplementary materials: Detailed guidance on benchmark survey questions for evaluating socially responsible research progress at the individual researchers level. (This dataset provides a detailed account of indicators that can be used to measure RRI progress around the world against the UNESCO Recommendation for Science and Scientific Researchers at the level of individual researchers). Data are available under the terms of the Creative Commons Attribution 4.0 International Public License (CC-BY 4.0).
