# Supplementary material for: Global indicators framework for socially responsible research and innovation (RRI): Aligning standards to monitor public and researcher perspectives with the UNESCO Recommendation on Science and Scientific Researchers
Source: Open Res Eur. 2023 Aug 2;2:36. Originally published 2022 Mar 10. [Version 2] doi: 10.12688/openreseurope.14232.2 (PMC10446147; doi:10.12688/openreseurope.14232.2)
Supplement: Peer review history

# Peer review: Reviewer response for version 2

**DOI:** 10.21956/openreseurope.17667.r34200

**Published:** 2023-08-31

**Authors:** Sara Wilford, Mayen Cunden, Tonii Leach

**Affiliations:** 1 DeMontfort University, Leicester, UK

The author has decided not to address any of the comments raised in the extensive report we wrote previously (as reviewer 2) by stating that it would be a different paper should they do so. We are therefore unable to comment on the amendments as nothing we raised has even been commented on, changed or revised. In light of this, our view also does not change.

Is the argument persuasive and supported by appropriate evidence?

No

Is the work clearly and cogently presented?

No

Is the topic of the essay discussed accurately in the context of the current literature?

No

Does the essay contribute to the cultural, historical, social understanding of the field?

No

Reviewer Expertise:

All three reviewers work in the area of computer and technology ethics and have strong backgrounds in RRI.

We confirm that we have read this submission and believe that we have an appropriate level of expertise to state that we do not consider it to be of an acceptable scientific standard, for reasons outlined above.

# Peer review: Reviewer response for version 1

**DOI:** 10.21956/openreseurope.15350.r30259

**Published:** 2022-10-20

**Authors:** Penny Haworth

**Affiliations:** 1 South African Institute for Aquatic Biodiversity Penny Haworth (SAIAB), Grahamstown, South Africa

With regard to the presentation of a persuasive argument supported by evidence, the references supplied suggest a broad base of reading into, as well as some significant contributions to, the available literature on the topic. 

 As far as my personal knowledge of the current literature goes, discussion points appear well supported. 

 The final review question: 'Does the essay contribute to the cultural, historical, social understanding of the field?' is far-reaching in its focus and the debate is in a formative phase.

 As a reviewer who is not an academic, my knowledge of the discipline is limited and my opinion is based more on experiential learning and experience in various contexts since the early 1980s first as a teacher, then museum educator and in the past 18 years consolidating that experience and applying it as a science communicator. Historically, it appears that the period covered by this article goes back only as far as the UNESCO-led RSSR (2017), although some of the survey information used was gathered prior to this (2014). It depends how far reaching this essay wants to be in its historical scope, but it could be informative to remember that museums and science centres have been grappling with ways to improve public understanding of science through interpretative design and the use of suitable language in interactive displays and exhibitions at least since the late 1980s.

 Whilst the terminology has changed and RRI is now used (to a degree) to capture the idea of the social relevance and applicability of research and innovation, a return to some of the earlier literature regarding living museums and the development of interpretative frameworks to enable more effective engagement with pubic audiences may be useful in developing meaningful indicators for a broad spectrum of policies and practices.

 Similarly, whilst the essay mentions the need for "globally relevant and usable indicators", it does not make clear reference to indigenous knowledge systems (IKS), which in third world and developing countries are beginning to be sought out and integrated into research and innovation. Perhaps the integration of IKS into RRI and contributions of RRI policies and practices to sustainable development could become indicators in their own right.

Is the argument persuasive and supported by appropriate evidence?

Yes

Is the work clearly and cogently presented?

Yes

Is the topic of the essay discussed accurately in the context of the current literature?

Yes

Does the essay contribute to the cultural, historical, social understanding of the field?

Partly

Reviewer Expertise:

As stated in my report, as reviewer who is not an academic, my knowledge of the discipline is limited and my opinion based on experience in various contexts since the early 1980s as a teacher, museum educator and science communicator.

I confirm that I have read this submission and believe that I have an appropriate level of expertise to confirm that it is of an acceptable scientific standard.

# Peer review: Reviewer response for version 1

**DOI:** 10.21956/openreseurope.15350.r29465

**Published:** 2022-06-27

**Authors:** Sara Wilford, Mayen Cunden, Tonii Leach

**Affiliations:** 1 DeMontfort University, Leicester, UK

This paper does not ‘read well’ and jumps between ideas without discussion, preamble or context.  It is unclear how the paper goes beyond the state of the art despite the author’s assertions, and the current state of the art is not described or discussed. There is little discussion or critique, and the paper does not adequately discuss benchmark survey data in the context of making recommendations to evaluate processes that utilise RRI at its core. The abstract mentions a framework but does not provide a recognisable framework or approach within the paper itself. 

 The whole premise is the importance of evaluation of RRI practices and policies, but the paper fails to provide context, core principles of RRI or to reference key authors in the field such as von Schomberg, Owen, Stilgoe, Stahl, etc.

 A key criticism is that the author draws much of their perspectives from their own published work and therefore does not draw adequately on the wealth of discourse and literature currently available.

 The paper assumes that RRI should be deployed in a global context but provides little justification, and does not consider the global implications of implementing a fundamentally Euro-centric framework – given that the UNESCO RSSR referred to throughout this essay states an aim towards a set of ‘shared values’, it is difficult to reconcile this with the inference that those shared values should, by the nature of RRI, be European in nature. The essay does allude to challenges of different and competing national research agendas but offers no explanation of these differences nor competition/competitiveness.

 There are also some inconsistencies and inaccuracies. For example, the author states RRI is integral to the RSSR policy instrument and “linking RRI to the 2017 Recommendation”. However, later they take the position that the author-defined 10 key priority areas, “identified and agreed by UNESCO Member States” i.e., the RSSR Policy instrument, should be used to evaluate the development of RRI principles. However, Annex II, Item 7.4 of the agenda of the 37th UNESCO session, (Paris 2017), makes no mention of these 10 priority areas in its 48 recommendations over seven sections. Further, the enshrinement of ‘aspects’ of RRI in the RSSR is contestable as Annex II Recommendation on Science and Scientific Researchers of the General Conference of UNESCO (pp117-127), makes no specific mention of RRI. The basis for recognition or understanding of “the principles included in the RSSR,” by the public, (layman), deemed “not well established” is not justified, and the essay argues for an indicator framework to monitor or measure such a perspective, but does not indicate how such an exercise will offer “specific ways to improve on existing RRI indicators”.

 The paper is somewhat situated in current discourses of the literature although it takes a contrary position. For example, its advocacy for “good metrics to measure progress” contradicts, without argument or justification, a Global Research Council (2021) finding of “a shift away from reliance on metrics towards more qualitative or mixed-methods modes of assessment” amongst the global signatories to responsible research assessment ( GRC Publications | Global Research Council). Furthermore, the author’s recommendation for the use of “existing metrics” to save resources and improve quality is unfounded.  

 The essay engages with a number of surveys around the topic of RRI indicators, although it is not possible to access the linked data and the checklist icon does not highlight any questions and provides no guidance to the reader to parts of the document likely to be most relevant.

 The surveys take a diversity of approaches, but it is unclear how much overlap there is between the countries/regions covered, and whilst this is an important issue to address, without understanding which group/country/demographic each survey was tailored to, it is difficult to assess the use of these in the context of the 195 UNESCO RSSR signatories.

 From a preliminary view, it appears that European countries feature (somewhat unsurprisingly) heavily in the selected public opinion surveys on RRI progress, and the suitability and/or effectiveness of these surveys being translated globally is ill-explored here.

 Justification for the selection of the six sources of global survey data are absent, as are explanations of its relevance either to RRI or RSSR. Why just the six (at public level)?

 It is notable that whilst the WGM is relied upon as ‘truly global’, the author does not clarify that the 2020-21 survey covered a reduced scope of 113 countries, rather than the 140+ countries in the 2018 version – given 195 countries signed up to the UNESCO RSSR, this does not, in fact, feel ‘truly global’. Further, the use of the 2014 Eurobarometer report feels outdated, given the development of approaches to (and measures of) RRI since then.

 In the section on individual researcher level of measurement, the author fails to note that the UNESCO formal national reporting it is left up to the member states themselves to determine ‘the criteria for inclusion in the category of persons recognized as scientific researchers’, making global indicators for this category difficult to compare.

 The essay seeks to monitor public and researcher perspectives but makes no justifiable case for establishing the rationale. Equally, it is quite unclear as to the “progress” of what is monitored by the “high-quality indicators”: researcher perspectives or impacts of (the UNESCO) policy instrument or indeed its use to “benchmark the development of socially responsible research and innovation”.

 To pass peer review, this paper will require considerable revision to include a comprehensive review of current literature across all aspects of the paper’s topic, and to provide justification and explanation of the ideas presented. The paper should also be re-structured to provide a narrative that guides the reader through the paper, and provides evidence-based conclusions.

Is the argument persuasive and supported by appropriate evidence?

No

Is the work clearly and cogently presented?

No

Is the topic of the essay discussed accurately in the context of the current literature?

No

Does the essay contribute to the cultural, historical, social understanding of the field?

No

Reviewer Expertise:

All three reviewers work in the area of computer and technology ethics and have strong backgrounds in RRI.

We confirm that we have read this submission and believe that we have an appropriate level of expertise to state that we do not consider it to be of an acceptable scientific standard, for reasons outlined above.

# Response

**Published:** 2022-07-25

**Authors:** Eric Jensen

**Affiliations:** Institute for Methods Innovation, Ireland

Thanks for taking the time to provide such extensive feedback. The current structure of the article submission was adapted from its original form to follow mandatory instructions from the Open Research Europe editorial staff. This particular article type (essay) in this journal is for 'articles outlining an argument or personal point of view.' There is no expectation in the guidelines for any kind of literature review to be included, and indeed that was never intended here. The 10 key priority areas were officially adopted by the UNESCO Executive Board as a practical way forward for distilling the much longer original ratified text of the Recommendation on Science and Scientific Researchers. This was primarily aimed at making the monitoring process (which runs on a four-yearly cycle) more manageable for Member-States. The details are now available on the UNESCO website for the Recommendation on Science and Scientific Researchers, with more information coming over the next year or two.

# Peer review: Reviewer response for version 1

**DOI:** 10.21956/openreseurope.15350.r28820

**Published:** 2022-04-12

**Authors:** Łukasz Nazarko

**Affiliations:** 1 Bialystok University of Technology, Bialystok, Poland

Operationalising Responsible Research and Innovation paradigm is a necessary step towards the maturity of this concept. Some progress has already been made in this area - more with regard to public entities (research institutes, higher education institutions, funding agencies), less with regard to industry (enterprises, private labs).

 The submitted essay looks at the possibility of developing a framework (a set of indicators) to monitor progress toward the socially responsible innovation at the global level, from the perspective of the public and individual researchers. Author proposes to make use of the already existing vehicle of the 2017 UNESCO-led Recommendation on Science and Scientific Researchers (RSSR). As such, this proposal is rational and offers an approach that addresses a frequent problem of "reinventing the wheel".

 Since this is an essay, I recommend that the Introduction is supplemented with an explanation/definition of socially responsible research and innovation. The main body of the text consists of short descriptions of various data sources/surveys that, according to the author, may be linked to socially responsible research and innovation. The attempt to link particular surveys to specific aspects/dimensions of RRI is desirable. It would indicate clearly how a particular survey/dataset is linked to the concept of RRI. Moreover, some sort of a concise synthesis of all presented surveys would be helpful. What RRI dimensions are covered? What dimensions are not yet covered?

 The chosen form of the submission (essay) imposes a certain discipline on the author. One has to present thoughts in a coherent and non-trivial way, without pondering on details and nuances in order to keep the text short. The author has been successful to this end.

 Eight out of 13 referenced works are the author's own publications. That would be a problem with a research paper but is acceptable in an essay, especially given that the references are relevant.

Is the argument persuasive and supported by appropriate evidence?

Partly

Is the work clearly and cogently presented?

Yes

Is the topic of the essay discussed accurately in the context of the current literature?

Yes

Does the essay contribute to the cultural, historical, social understanding of the field?

Yes

Reviewer Expertise:

RRI, Technology Assessment, Innovation Policy

I confirm that I have read this submission and believe that I have an appropriate level of expertise to confirm that it is of an acceptable scientific standard.
